# NFkappaB is a Key Player in the Crosstalk between Inflammation and Cardiovascular Diseases

**DOI:** 10.3390/ijms20071599

**Published:** 2019-03-30

**Authors:** Antonella Fiordelisi, Guido Iaccarino, Carmine Morisco, Enrico Coscioni, Daniela Sorriento

**Affiliations:** 1 Department of Advanced Biomedical Sciences, Federico II University of Naples, 80131 Napoli, Italy; antonella.fiordelisi@gmail.com (A.F.); guiaccar@unina.it (G.I.); carmine.morisco@unina.it (C.M.); 2 Division of Cardiac Surgery, AOU San Giovanni di Dio e Ruggi d’Aragona, 84131 Salerno, Italy; coscionienrico@gmail.com

**Keywords:** inflammation, cardiovascular diseases, NFκB, GRK

## Abstract

Inflammation is a key mechanism of cardiovascular diseases. It is an essential component of atherosclerosis and a significant risk factor for the development of cardiovascular events. In the crosstalk between inflammation and cardiovascular diseases, the transcription factor NFκB seems to be a key player since it is involved in the development and progression of both inflammation and cardiac and vascular damage. In this review, we deal with the recent findings of the role of inflammation in cardiac diseases, focusing, in particular, on NFκB as a functional link. We describe strategies for the therapeutic targeting of NFκB as a potential strategy for the failing heart.

## 1. Inflammation in Cardiovascular Diseases

Emerging evidence suggests that chronic inflammation participates in the development and progression of cardiovascular diseases (atherosclerosis, aortic valve disease, myocardial infarction, heart failure) and cardiometabolic disorders (obesity, insulin resistance) [1,2,3,4,5,6,7,8]. Equally important, several pro-inflammatory markers are elevated in patients with cardiomyopathies and have been correlated with the prognosis and severity of disease [2] suggesting a potential relationship between markers of inflammation and risk of future cardiovascular events [9]. 

### 1.1. Inflammation and Atherosclerosis

Atherosclerosis is a multi-step process that consists of four main phases: endothelium injury; foam cell formation; smooth muscle cell proliferation; formation and rupture of the atherosclerotic plaque. Both adaptive and innate immunity is involved in the development and progression of atherosclerosis [10]. Indeed, the adhesion molecules and chemokines in the early phases of the process promote the recruitment of both monocytes/macrophages and T cells. In particular, inflammatory cells are recruited from the circulation and migrate into activated endothelial cells. The endothelial migration is mainly mediated by cellular adhesion molecules such as vascular-cell adhesion molecule 1 (VCAM-1), which is typically up-regulated in response to hypercholesterolemia [11]. Through the chemo-attractant cytokine, MCP-1, leukocytes are recruited to the site of the lesion [12]. Once resident in the intima, the monocytes differentiate into macrophages that bind oxidized lipoproteins giving rise to the arterial foam cell (a hallmark of the arterial lesion). This latter secretes pro-inflammatory cytokines (IL-1β, IL-6, IL-8, TNFα, TGFβ) that amplify the local inflammatory response, induces reactive oxygen species production and recruits VSMC to the site of the lesion. T cells in the lesion (Th1 subtype) induce the production of IFNγ, IL-2, TNFα which, in turn, cause the activation of macrophages and vascular cells and further promote inflammation [13]. Thus, inflammation clearly plays a key role in the atherosclerotic process since inflammatory cells are key effectors in its early phases, cytokines accelerate the progression of the lesions leading to plaque rupture, thrombosis and the clinical manifestation of the acute coronary syndrome [14]. 

Experimental studies in pre-clinical models suggest potential molecular mechanisms by which cytokines exert their effects. It is known that Ang II has pro-inflammatory effects in the vascular wall by inducing ROS production and NFκB activation which in turn induces the expression of cytokines, chemokines, and adhesion molecules, including IL-6, MCP-1 and VCAM-1 [15,16,17]. The interaction of the proinflammatory cytokine IL-6 with the renin-angiotensin system (RAS) has been suggested as an important pathogenetic mechanism in the atherosclerotic process [18]. Indeed, IL-6 induces oxidative stress and endothelial dysfunction in a model of atherosclerosis by activating the angiotensin II type 1 receptor and these effects are absent in transgenic mice with the deletion of AT1 receptor [18]. It has also been suggested that IL-6 and MCP-1 secretion in response to ANG II are actually codependent and accelerates macrophage-mediated vascular inflammation [19].

The inflammatory process in atherosclerosis leads to increased blood levels of inflammatory cytokines and other molecules which are considered useful biomarkers of cardiac dysfunction. Indeed, the levels of C-reactive protein and interleukin-6 are elevated in patients with unstable angina and myocardial infarction and it has been shown that high levels are associated with a worse prognosis [20,21].

### 1.2. The Role of Inflammation in Myocardial Infarct and Heart Failure

**Figure 1:**
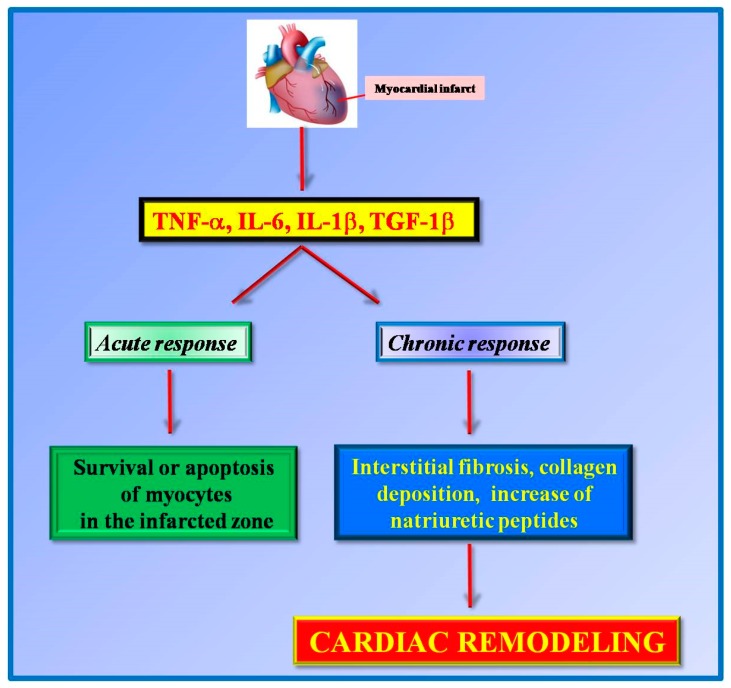
Pro-inflammatory cytokines (TNFα, IL-6, IL1β, TGF-1β) are released in response to myocardial infarction. These cytokines exert different effects on acute and chronic responses. In an early phase, they exert a cardioprotective role by regulating the survival of cardiomyocytes in the infarcted area while in chronic responses these cytokines favor cardiac remodeling.

In response to the insults, several molecular mechanisms got activated in the heart to initiate the structural remodeling of the organ. This process involves both cardiac and non-cardiac cells, including inflammatory cells [22]. For instance, in response to acute myocardial infarction, there is an early increase of proinflammatory cytokines, such as TNF-alpha, IL-6, IL-1beta, and transforming growth factor 1-beta (TGF-1beta). The acute release of these inflammatory cytokines initially regulates the survival or apoptosis of myocytes in the infarcted zone [23]. However, in a later phase, the continuous increase of these cytokines production promotes interstitial fibrosis and collagen deposition in the non-infarcted zone leading to ventricle dysfunction [23,24]. Cytokines induce the activity of local matrix metalloproteinase (MMP2 and MMP9) in the infarcted area and the expression of natriuretic peptides (ANP and BNP) favoring cardiac remodeling [25]. These findings support the idea that inflammation exerts a cardioprotective role in the acute responses and a deleterious effect in the chronic responses (Figure 1). 

At the molecular level, the upregulation of cytokines in the heart activates intracellular pathways which significantly affect cardiac cell biology: activation of transcription factors such as NFκB and AP-1 [26], cardiomyocytes survival and apoptosis [27], ROS production [28], and cardiac cell contractility [29]. The link between cytokines and oxidative stress is evident from the study of Machida and colleagues [28]. Indeed, in transgenic mice with overexpression of TNFα, the production of hydroxyl radical is increased and, accordingly, the antioxidant capacity of MnSOD is impaired [28]. Additionally, TNFα induces mitochondrial ROS production in cardiac myocytes [30] that is associated with mitochondrial DNA damage favoring the progression of heart failure [31]. 

Besides the effects on oxidative stress, cytokines such as TNFα and IL-6, are able to attenuate myocyte contractility in both direct and indirect manners. Indeed, TNFα directly reduces sarcoplasmic reticulum (SR) calcium uptake through the inhibition of SR calcium adenosine triphosphatase expression and activity [29] and this effect is rescued by the removal of cytokine exposure [32]. Furthermore, TNFα decreases myocyte contractility indirectly by inducing nitric oxide synthesis in isolated myocytes thus attenuating myofilament calcium responsiveness [23,33]. 

Additionally, IL-1β has been shown to affect cardiac function by activating NO synthase activity in cardiac myocytes [34] which in turn lowers energy production and myocardial contractility via impairment of mitochondrial function [35]. Accordingly to these in vitro findings, targeting these pro-inflammatory cytokines could significantly affect the clinical outcomes of heart diseases, supporting the entangled role of inflammation in cardiovascular events through the regulation of mitochondrial function [36,37].

### 1.3. Inflammation and Cardiovascular Risk

An association between inflammation and cardiovascular risk has been suggested by the evidence that inflammatory cytokines, including IL-1β, IL-6, IL-18, and TNF-α, are increased in patients with heart failure, and increased inflammatory markers, such as C-reactive protein, predict a worse survival during acute coronary syndromes [36,38,39]. Increased levels of proinflammatory cytokines in response to myocardial damage are important prognostic factors, correlating with increased mortality rates in HF patients [40].

Moreover, the incidence of cardiovascular events is increased in patients affected by chronic inflammatory disease, such as Rheumatoid Arthritis (RA) and might sustain the increased risk of cardiovascular events in this population. Interestingly, though, in RA it is possible to observe structural abnormalities both at cardiac (increase ventricular mass, intraventricular septum end-diastolic thickness, LV posterior wall end-diastolic thickness, and aortic root diameter) and vascular (arterial stiffness, increased intima-media thickness of the carotid arteries) levels. These abnormalities may be due to the systemic effects of the immune system [41,42,43].

Accordingly, anti-inflammatory therapies reduce the risk of cardiovascular events in patients with rheumatoid arthritis, as demonstrated in large prospective cohort studies [44,45] in RA patients treated with methotrexate. Furthermore, in RA patients the selective inhibition of TNF-α by means of infliximab and etanercept or the inhibition of IL-6 by tocilizumab exerted a positive effect on endothelial function [46]. Also, pre-clinical studies confirmed the atherogenic role of immune cells in rheumatoid arthritis that is associated with elevated levels of inflammatory cytokines [47,48]. 

TNF-α, for instance, upregulates the expressions of adhesion molecules and chemokine, favoring the recruitment of monocytes to the lesions, and also increases SRA expression in macrophages favoring the uptake of oxidized LDL.

## 2. Targeting Inflammatory Signaling in Cardiovascular Diseases

The identification of specific cytokines which affect different intracellular signaling pathways in response to cardiac damage supported the idea that targeting these cytokines could ameliorate cardiac responses. Several pieces of evidence (study with monoclonal antibodies against the IL-1β and IL-6 receptor) support this hypothesis. Moreover, the evidence that the cardiovascular therapy (statins, phosphodiesterase inhibitors, antiarrhythmic, COX-2 inhibitors, Angiotensin II receptor blockers) also modulates cytokines expression and inflammatory signaling sustain the proof of concept that inflammation has a functional role in cardiovascular diseases.

### 2.1. The Modulation of Inflammatory Proteins Expression

Given the contribution of inflammation in the development of atherosclerotic plaque, different treatments have been tested which specifically act on inflammatory signaling [49]. These include the phospholipase A2 inhibitors, which act on atherogenic lipoproteins and foam cells [50], and some general antioxidants, that may affect inflammatory pathways at different steps of the atherosclerotic process [51]. Furthermore, statins, which are generally used to reduce cholesterol synthesis, have been shown to exert anti-inflammatory action independently from lipid-lowering effects [52,53]. Indeed, the treatment with statins reduces the expression of vascular cell adhesion molecule 1 and intracellular adhesion molecule 1 in human endothelial cells in vitro [54]. Accordingly, statins reduce the growth of macrophages and lowered the metalloproteinase activity to stabilize atherosclerotic plaques in vivo [55]. In animal models of atherosclerosis, statins reduce the inflammatory infiltrate in the arterial wall and the expression of different inflammatory cytokines [56]. Statins are also known to positively affect the outcomes in chronic heart failure [57] by improving cardiac function, and decreasing plasma concentrations of TNF-α, IL-6, and brain (B-type) natriuretic polypeptide [58]. 

Additionally, phosphodiesterase inhibitors, which are used for the treatment of heart failure, are able to reduce the production of cytokines. In particular, pimobendan has been shown to decrease the production of intracardiac IL-1β, IL-6, TNF-α and nitric oxide [59] by inhibiting the activation of NFκB [60] in mouse models of heart failure. Accordingly, in clinic pimobendan seems to improve the quality of life and to decrease the number of cardiovascular events in patients with heart failure [61]. In the same manner, amiodarone, an antiarrhythmic drug that improves the long-term prognosis of heart failure, has been shown to inhibit the production of TNF-α and IL-6 [62,63]. These findings suggest that the anti-inflammatory properties of this drug contribute to its beneficial cardiovascular effects. 

Hypertension is among the main risk factor for developing cardiovascular diseases and, in particular, of atherosclerotic vascular diseases. Chronic inflammation causes the increase of oxidative stress, CRP synthesis, and pro-inflammatory cytokines production and this is associated with endothelial dysfunction in hypertension. It has been shown that anti-inflammatory treatments have also beneficial effects on vascular function. Indeed, COX-2 inhibition, by means of celecoxib, reduces CRP levels in patients with both severe CAD [64] and arterial hypertension [65] associating with an improved endothelial function. Additionally, the treatment with Angiotensin II receptor blockade reduces the levels of CRP, TNFα, IL-6, and MCP-1 in patients with essential hypertension [66].

### 2.2. The Therapeutic Monoclonal Antibodies

Given the role of inflammation in several diseases, blocking the activity of the specific cytokine that is involved in the pathogenesis of the condition could provide an additional advantage over standard therapy. In this context, a novel technology, the therapeutic monoclonal antibodies (mAbs), has been proposed and it is now widely accepted to treat cancer, autoimmune and infectious diseases. Recently, this strategy has also been extended to the cardiovascular field and, in particular, the effectiveness of mAbs against specific inflammatory cytokines was tested in clinical trials. The CANTOS trial, for instance, involved over 10,000 patients with a previous myocardial infarction and high levels of C-reactive protein. Patients were treated with Canakinumab, a monoclonal antibody targeting IL-1β, at different doses [67]. This study shows that Canakinumab at a dose of 150 mg every 3 months lowers the rate of recurrent cardiovascular events compared to the placebo. Since it is known that Interleukin-1β strongly induces IL-6 production by many cell types including vascular endothelial and smooth muscle cells [68], it has also postulated that the ameliorated cardiovascular outcomes to canakinumab treatment could be mediated through the IL-6 signaling pathway. Indeed, canakinumab dependent reduction of IL-6 was associated with the reduction of cardiovascular events [69]. Additionally, in a randomized, controlled trial in regarding 100 patients with N-STEMI, a single dose of Tocilizumab, a humanized anti-IL-6 receptor antibody, prior to coronary angiography, attenuated the inflammatory response [70]. In parallel to the CANTOS study, the CIRT study was performed to evaluate the effectiveness of an alternative approach to inflammation inhibition (methotrexate) in patients with previous myocardial infarction or multivessel coronary disease who also had either type 2 diabetes or metabolic syndrome [71]. Patients were treated with Methotrexate, an inhibitor of folic acid, given the effectiveness of this anti-inflammatory drug in patients with RA. However, low doses of methotrexate failed to prevent cardiovascular events and to reduce IL-1b, IL-6 and CRP levels [71], being in contrast with CANTOS study. However, it should be taken into account that inclusion criteria for patients enrollment were different since CIRT did not screen for CRP levels while CANTOS enrolled patients with high levels of CRP. Moreover, the therapeutic targets are also different underlining the importance of specific inflammatory signaling in the development of cardiac events and the effectiveness of therapeutic monoclonal antibodies compared with generic anti-inflammatory drugs. 

The complexity of the role of inflammation in cardiovascular diseases is testified by the failure of a number of trials showing that monoclonal antibodies raised against specific cytokines worsened prognosis. Indeed, data from a clinical trial show that in patients with heart failure the treatment with Infliximab, a chimeric monoclonal antibody to TNF-α, or Etanercept, a soluble TNF receptor, increased mortality [72]. Thus, the beneficial effects of therapeutic treatments for heart failure could be just in part due to the modulation of inflammatory responses but the best treatment is the one which affects both inflammation and cardiac damage mechanisms. 

## 3. Molecular Mechanisms Involved in Inflammatory Responses: the Role of NFκB

Different molecular mechanisms activate inflammatory responses leading to cytokine production and release. The type of cytokine and its specific effect differ during an inflammatory response depending on tissues and specific physio-pathological condition. However, a common feature of inflammatory intracellular signaling is the activation of specific transcription factors which drive the expression of cytokine production. Among them, NFκB is the best-known director of this phenomenon. NFκB is a ubiquitous transcription factor involved in cellular responses to stimuli such as stress, cytokines, free radicals, ultraviolet irradiation, oxidized LDL, and bacterial or viral antigens [73,74,75,76,77]. 

The family of NFκB proteins includes p52/p100, p50/p105, c-Rel, RelA/p65, and RelB. These proteins function as dimeric transcription factors that regulate the expression of genes thus affecting a broad range of biological processes. In the canonical pathway, NFκB is bound and inhibited by IκB proteins. Proinflammatory cytokines, LPS, growth factors, and antigen receptors activate an IKK complex (IKKβ, IKKα, and NEMO), which phosphorylates IκB proteins inducing their ubiquitination and proteasomal degradation. NFκB is, therefore, free to translocate to the nucleus where alone or in combination with other transcription factors (AP-1, Ets, and Stat) induces target gene expression [78]. In the non-canonical pathway, p100/RelB complexes are inactive in the cytoplasm. After receptor activation, including LTβR, CD40, and BR3, the kinase NIK is activated inducing IKKα complexes. These latter phosphorylate p100 that is proteolytically processed to p52 which, together with RelB complexes, translocate to the nucleus and induce target gene expression [78].

The activation of NFκB depends on different stimuli. In the heart, both innate and adaptive immunity are involved in responses to tissue injury due to pathogen-associated molecular patterns (PAMPs) or damage associated molecular patterns (DAMPs) which stimulate specific membrane receptors pattern recognition receptors (PRRs) [79]. Many PRRs triggered by PAMPs and DAMPs initiate a signaling cascade which culminates into the activation of NFκB, beyond the activator protein 1, the interferon regulatory factors transcription factor and the inflammasome [79]. 

The pathogenetic role of NFκB has been demonstrated in many diseases from cancer to cardiovascular diseases [80,81,82], where it exerts a double effect: regulation of immunity by driving the expression of genes involved in inflammation [83] and regulation of the expression of specific target genes involved in the progression of the pathology. This suggests that NFκB could be a potent therapeutic target in those pathologies which are characterized by the elevated activity of this transcription factor and for which inflammation favors organ damage, such as in cardiovascular diseases.

## 4. NFκB in Cardiovascular Diseases

Several reports show that the transcription factor NFκB, known to regulate the expression of inflammatory cytokines, also activates genes involved in various cardiovascular diseases, in the pathogenesis of cardiac remodeling and heart failure [84,85,86]. NFκB is activated in the heart in many conditions: during acute ischemia and reperfusion [87,88,89], during unstable angina [86,90] or in response to preconditioning [91]. In patients with heart failure, for instance, NFκB is activated both in cardiomyocytes [92] and in peripheral white blood cells [93]. However, its role in these pathological conditions still remains unknown. Several studies suggest that NFκB is cardioprotective during acute hypoxia and reperfusion injury through the inhibition of BNIP-3 expression [94]. However, in cardiac remodeling, the prolonged activation of NFκB is cytotoxic and promotes heart failure by triggering a chronic inflammatory response [95]. These controversial results confirm that NFκB signaling is a very complex process involving several components and at multiple steps of regulation. In this complexity, timing and cellular context are critical factors for the NFκB effect.

The proof of concept that this transcription factor is involved in the development and progression of heart diseases comes from pre-clinical studies. In a mouse model with a cardiomyocyte-specific expression of a constitutively active form of IKK2, hearts showed inflammatory infiltrate, fibrosis and atrophy of myocytes [96]. The expression of IκBα super-repressor, which inhibits NFκB, prevented the development of the disease suggesting that this phenomenon is dependent on NFκB activation [96]. Accordingly, 4 weeks after coronary ligation, transgenic mice with the cardiac-selective overexpression of the IκBα super-repressor showed improved survival, chamber remodeling, systolic function, and pulmonary congestion, associated with a reduction of NFκB p65 activation, cytokine expression, fibrosis, and apoptosis [97]. NFκB is also a fine regulator of cardiac hypertrophy. Indeed, cardiac-specific deletion of p65 in mice decreases the hypertrophic response in response to pressure overload stimulation, leading to a reduction of pathological remodeling and the rescue of contractile function [98]. In this model of cardiovascular disease, the interaction between NFκB and the nuclear factors of activated T cells (NFAT) has been hypothesized. Additionally, the inhibition of NFκB using direct gene delivery of sh-p65-RNA results in regression of cardiac hypertrophy [99]. 

## 5. Targeting NFκB in the Failing Heart: the Role of GRKs

As described above, the anti-inflammatory treatment alone resulted not enough to revert cardiac damage [72] but drugs for the treatment of cardiovascular diseases that also have anti-inflammatory properties were more efficient acting on both inflammation and cardiac damage [100,101,102,103]. Thus, for the development of a novel effective therapeutic strategy, it is essential to find a potential target that is involved in both processes. In this context, NFκB seems to be a good candidate since it regulates both cytokine production and hypertrophic genes expression. 

**Figure 2:**
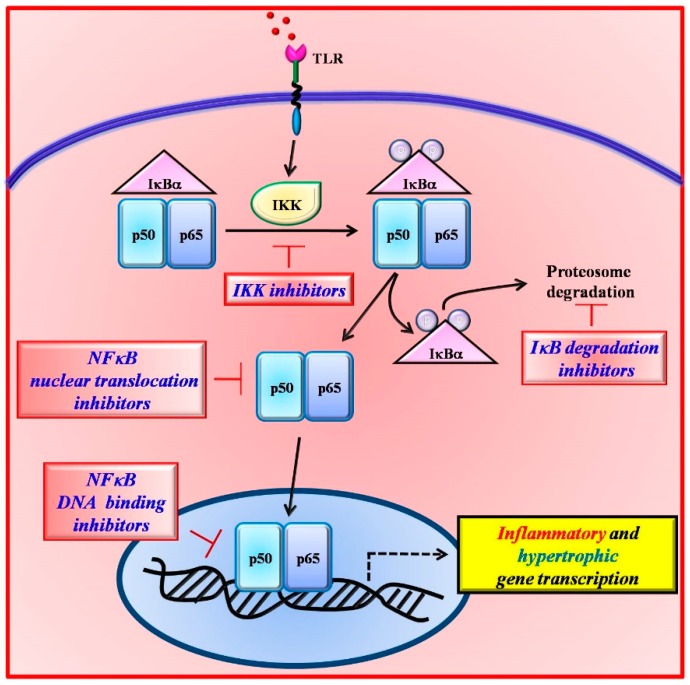
The inhibition of NFκB signaling can occur at different steps of its activation pathway. Several inhibitors have been designed which act on IκBα degradation (TP-110), IKK activity (EF-24), NFκB nuclear translocation (SN-50), and DNA binding (tacrolimus).

In the last decades, several inhibitors of NFκB have been developed, which act at different steps of its intracellular signaling pathway [104,105] (Figure 2), including anti-oxidants [106,107], proteasome and proteases inhibitors [108,109], inhibitors of IκBα [110,111], and miscellaneous inhibitors [112,113]. These compounds, although inhibiting NFκB, showed also toxic effects. The proteasome inhibition, for example, blocks the degradation of all ubiquitinated proteins within the cell greatly affecting cell biology and causing severe side effects. Therefore, most of them failed transferability to the clinical scenario. Therefore, there is a need for the identification of novel therapeutic relevant targets of NFκB signaling.

Besides their well-known effects in failing cardiac cells [114,115,116,117,118], the involvement of G Protein-Coupled Receptor Kinases (GRK) in inflammatory processes has been recently described [119,120,121]. Several observations suggest that GRKs play a critical role in different inflammatory disorders and in autoimmune diseases such as multiple sclerosis and rheumatoid arthritis. Indeed, in peripheral blood, mononuclear cells collected from patients with autoimmune diseases, GRK2 and GRK6 are significantly reduced [122,123] and this is associated with an increased GPCRs downstream signaling (enhanced cAMP and reduced TNF-α production) [124]. Actually, three members of the GRKs family (GRK2, GRK5, and GRK6) have been shown to affect inflammatory responses through the specific regulation of NFκB signaling [125,126,127]. 

GRK2 affects NFκB signaling by interacting with IκBα and p105 [127]. GRK2 phosphorylates IκBα in response to TNFα in a macrophage cell line and in HEK293 cells [128]. Furthermore, GRK2 interacts with p105 thus inhibiting the ERK pathway in primary macrophages [129]. In neonatal rat cardiac fibroblasts, NFκB activation and IL6 levels were increased in response to arginine vasopressin, thus linking GRK2 to inflammation in response to cardiac stress [130]. Conversely, NFκB regulates GRK2 expression during inflammation [121]. 

GRK5 regulates NFκB signaling both in a kinase-dependent and independent manner. Indeed, it can directly phosphorylate NFκB p105, thus inhibiting LPS-dependent ERK activation in macrophages [126]. Furthermore, GRK5 interacts with IκBα through means of the RH domain and inhibits its degradation leading to the inhibition of NFκB activity in endothelial cells [131]. 

In response to TNF-α, GRK6 directly phosphorylates IκBα and induces its degradation by the proteasome. This promotes NFκB transcriptional activity and increases inflammatory responses [132]. 

**Figure 3:**
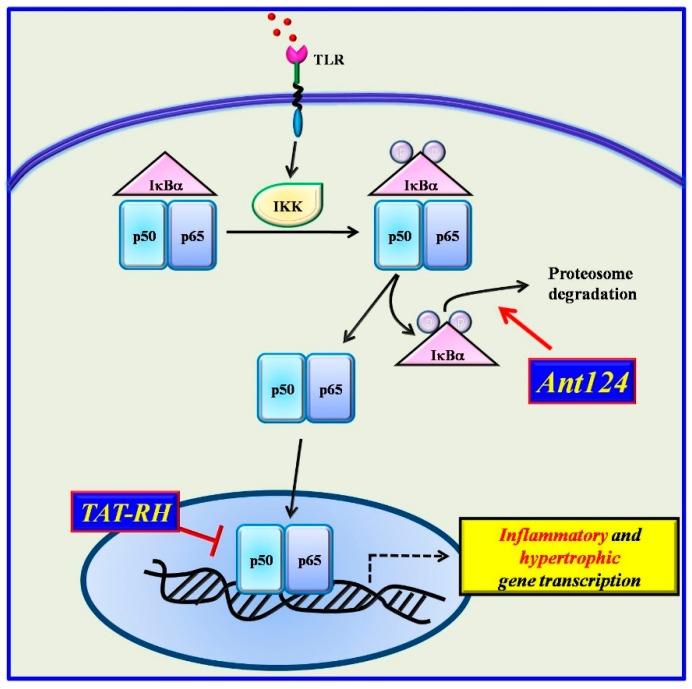
Two novel inhibitors have been designed based on the structure of GRKs. Ant124, which reproduces the HJ loop of GRK2, inhibits NFkB activation by blocking IκBα degradation. TAT-RH, which reproduces the RH domain of GRK5, binds IκBα and blocks the complex IκBα/NFκB in the nucleus thus preventing NFκB activation.

Given the involvement of GRKs in both inflammatory and cardiac diseases and their ability to regulate NFκB, several inhibitors have been designed on the GRKs sequence [115]. Among them, two specific compounds resulted to be effective inhibitors of NFκB in the heart: a peptide which reproduces the RH domain of GRK5 (TAT-RH) and the inhibitor of GRK2 kinase activity (Ant-124) (Figure 3).

### 5.1. The RH Domain of GRK5

It has been recently demonstrated that GRK5 regulates the transcriptional activity of NFκB [131]. In particular, in endothelial cells, GRK5 is able to bind the inhibitory protein of NFκB, IκBα, by means of the RH domain (GRK5-RH) and to stabilize the complex IκBα/NFκB in the nucleus, thus inhibiting NFκB transcriptional activity [131]. Indeed, GRK5-RH overexpression, by interacting with IκBα/NFκB complex, inhibits the transcriptional activity and DNA binding of NFκB both in the basal condition and after stimulation with LPS. GRK5-RH was shown to inhibit all NFκB dependent phenotypes, such as TNF-alpha transcription, endothelial cell migration and vascular tube formation in vitro and regenerative responses in vivo. Given these features of GRK5-RH, its effects were evaluated in different pathologies characterized by the elevated activity of NFκB: cardiac hypertrophy [133] and cancer [134]. In particular, in cardiomyoblasts, GRK5-NT inhibits phenylephrine-induced transcription of both NFκB and atrial natriuretic factor promoters. In vivo, its effect was evaluated in two different animal models of left ventricular hypertrophy: the spontaneously hypertensive rat and the normotensive Wistar Kyoto rat exposed to the chronic administration of phenylephrine. Intracardiac injection of an adenovirus encoding for GRK5-NT reduced cardiac mass in spontaneously hypertensive rats and prevented the development of phenylephrine-induced LVH in Wistar Kyoto rats. This was associated with the inhibition of NFκB transcriptional activity and its associated phenotypes (fibrosis and apoptosis) [133]. 

Given the notion that the adenoviral-mediated gene therapy seems to be not efficient for clinical application due to the toxicity and immunogenicity induced by the adenovirus itself, a synthetic protein reproducing the only RH domain of GRK5 (TAT-RH), engineered to be actively transported into the cells by means of the TAT domain without the support of other vehicles, has been developed. TAT-RH was tested in cancer cells showing its ability to inhibit NFκB transcription and induce apoptosis, reduce tumor angiogenesis, block cell proliferation and consequently tumor growth in a dose-dependent manner [134]. Successively, a minimum effective sequence of TAT-RH was designed reproducing only 10 amino acids of the RH sequence (RH10) that is effective to inhibit cancer growth and reduce oxidative stress [135]. RH10 has not been tested in the cardiovascular field yet, but these findings are promising of the RH10 effectiveness in cardiovascular diseases too. Compared with the other NFκB inhibitors, this strategy is based on a steric interaction of RH10 and IκBα and therefore does not require the inhibition of a general cellular mechanism such as the proteasome, suggesting that the side effects of chronic treatment with RH10, or a *small molecule* resembling it, will be of minimal intensity. 

### 5.2. Synthetic Inhibitors of GRK2

Similarly to GRK5, GRK2 is able to phosphorylate IκBα, even if with a lower affinity [136]. Such ability allows the kinase to regulate NFκB signaling in pathological conditions. Indeed, in cardiomyoblasts, the overexpression of GRK2 increased phenylephrine-dependent hypertrophic gene expression, and this was associated with an increase of the NFκB transcriptional activity [84]. The kinase-dead mutant of the kinases exerted the opposite effect, suggesting that GRK2 could regulate hypertrophy through the upregulation of NFκB activity in a phosphorylation-dependent manner. Thus, the inhibition of GRK2 activity could ameliorate cardiac function in response to hypertrophic stimuli. A synthetic peptide inhibitor of GRK2 was designed based on the catalytic domain sequence of GRK2 and conjugated with the antennapedia internalization sequence (Ant124). This peptide effectively inhibits the GRK2 catalytic activity and the phenotypes associated with GRK2 activation [137]. In two different in vivo models of left ventricle hypertrophy (LVH), the selective inhibition of GRK2 activity by Ant124 prevented hypertrophy and reduced the NFκB transcription activity [84]. Such results strongly support the idea that the inhibition of GRK2 could be an effective therapeutic strategy to both ameliorate cardiac function and reduce inflammatory responses which are associated with the excessive activity of NFκB.

## 6. Conclusions

Inflammation plays a key role in the development and progression of cardiovascular diseases as demonstrated by its involvement in atherosclerotic processes and its association with increased risk for cardiovascular events. Furthermore, several clinical trials demonstrate the effectiveness of anti-inflammatory treatment in the management of cardiovascular diseases. In this context, the transcription factor NFκB could represent a functional bridge between inflammation and cardiac pathologies. Indeed, the release of cytokines from activated macrophages which infiltrate the cardiac tissue induces the activation of NFκB also in the cardiomyocyte leading to hypertrophic gene expression and cardiac damage. The effectiveness of GRK-dependent inhibition of NFκB suggests that it could represent a potential therapeutic target for the treatment of cardiac dysfunction and the associated inflammatory phenotype. However, it should be considered that the systemic delivery of NFκB inhibitors could induce side effects by affecting other non-target organs. Thus, in the future, strategies for tissue-specific targeting of such inhibitors (i.e., the use of nanotechnology) should be developed to avoid potential side effects.
